# Systematic exploration of predicted destabilizing nonsynonymous single nucleotide polymorphisms (nsSNPs) of human aldehyde oxidase: A Bio‐informatics study

**DOI:** 10.1002/prp2.538

**Published:** 2019-11-22

**Authors:** Catarina Coelho, Jayaraman Muthukumaran, Teresa Santos‐Silva, Maria João Romão

**Affiliations:** ^1^ UCIBIO Chemistry Department Faculdade de Ciências e Tecnologia Universidade NOVA de Lisboa Caparica Portugal

**Keywords:** aldehyde oxidase, computational genomic, in silico analysis, pharmacogenetic, single nucleotide polymorphism

## Abstract

Aldehyde Oxidase (hAOX1) is a cytosolic enzyme involved in the metabolism of drugs and xenobiotic compounds. The enzyme belongs to the xanthine oxidase (XO) family of Mo containing enzyme and is a homo‐dimer of two 150 kDa monomers. Nonsynonymous Single Nucleotide Polymorphisms (nsSNPs) of hAOX1 have been reported as affecting the ability of the enzyme to metabolize different substrates. Some of these nsSNPs have been biochemically and structurally characterized but the lack of a systematic and comprehensive study regarding all described and validated nsSNPs is urgent, due to the increasing importance of the enzyme in drug development, personalized medicine and therapy, as well as in pharmacogenetic studies. The objective of the present work was to collect all described nsSNPs of hAOX1 and utilize a series of bioinformatics tools to predict their effect on protein structure stability with putative implications on phenotypic functional consequences. Of 526 nsSNPs reported in NCBI‐dbSNP, 119 are identified as deleterious whereas 92 are identified as nondeleterious variants. The stability analysis was performed for 119 deleterious variants and the results suggest that 104 nsSNPs may be responsible for destabilizing the protein structure, whereas five variants may increase the protein stability. Four nsSNPs do not have any impact on protein structure (neutral nsSNPs) of hAOX1. The prediction results of the remaining six nsSNPs are nonconclusive. The in silico results were compared with available experimental data. This methodology can also be used to identify and prioritize the stabilizing and destabilizing variants in other enzymes involved in drug metabolism.

**Abbreviations**
dbSNP: data base for single nucleotide polymorphism
INPS: impact of non‐synonymous mutations on protein stability
mCSM: mutation cutoff scanning matrix
NCBI: National centre for biotechnology information
PolyPhen: polymorphism phenotyping
PROVEAN: protein variation effect analyzer
SDM: site directed mutator
SIFT: sorting intolerant from tolerant

## 1 INTRODUCTION

SNPs are modifications occurring in a specific region of the genome, on a single nucleotide. There has been great interest in SNPs discovery since these are responsible for the majority of the genetic variations among the human population, and are expected to facilitate large‐scale genetic association studies.^1^ SNPs in coding regions are either synonymous, if they do not affect the protein sequence, or nonsynonymous (nsSNP) if they change the amino acid sequence of the codified protein. The nsSNP can be further divided into missense, if the nucleotide modification gives rise to a different amino acid residue, and nonsense, when the point mutation results in a premature stop codon that leads to a truncated form of the protein. Although the majority of the nsSNPs are phenotypically neutral, some of them can alter the structure and function of a protein, leading in some cases to disease associated conditions.^2^, ^3^, ^4^

**Figure 1:**
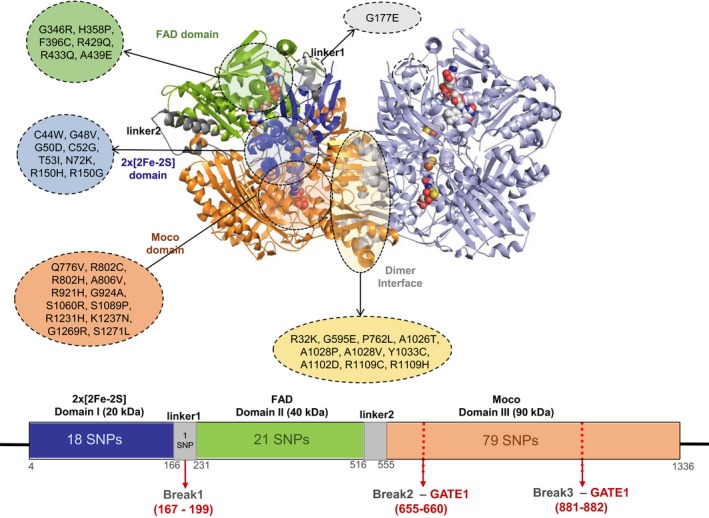
Overall representation of the hAOX1 homodimer and location of the most relevant nsSNPs in the hAOX1 structural domains. Domains I, II, and III are colored in blue, green, and orange, respectively

Aldehyde oxidase (AOX) belongs to the XO family of mononuclear molybdenum enzymes and is mainly involved in the metabolism of drugs and xenobiotic compounds. The human enzyme (hAOX1) is mainly expressed in the liver as a homo‐dimer, and each 150 kDa monomer consists of three different domains: the small *N*‐terminal domain I (20 kDa) with two spectroscopically distinct [2Fe‐2S] clusters; the central FAD domain II (40 kDa); and the *C*‐terminal catalytic domain III (90 kDa) which encloses the molybdopterin cofactor (Moco) (Figure 1). Two linker regions (linker1 and linker2) are responsible for connecting domain I to II, and domain II to III, respectively (Figure S1). The true physiological function of hAOX1 is unclear but it is known to be responsible for the failure of several phase I clinical trials, due to its diverse catalytic activity that include oxidations, hydrolysis of amides, and reductions.^5^, ^6^, ^7^, ^8^, ^9^ The interest in hAOX1, as a drug‐metabolizing enzyme, has increased in the past decade since its activity affects the metabolism of different drugs and xenobiotics, some of which designed to resist other metabolizing enzymes (eg cytochrome P450 monooxygenase isoenzymes). In the 1990s, studies using human liver extracts showed variations in the oxidation of known hAOX1 substrates, such as N1‐methylnicotinamide and benzaldehyde,^10^ and since then, differences in hAOX1 activity have been attributed to factors such as gender, age, cigarette smoking, drug usage, and disease states.^7^

Although more than 700 SNPs are reported in dbSNP,^11^ only few of them were studied in detail. Smith and coauthors^12^ suggested that N1135S polymorphism affects the metabolism of azathioprine, which might lead to nonresponse in the treatment of inflammatory bowel disease. Moreover in an independent study of the functional characterization of variants allowed the classification of the individuals mainly as fast‐metabolizers, poor‐metabolizers and individuals with no effect on the catalytic efficiency of hAOX1.^13^ Recently, Foti et al showed, using biochemical assays, that hAOX1 activity is not altered for some nsSNPs (S1271L, H363Q, A437V, L438V), while in other cases, the protein i) is unstable and cannot be produced (C44W); ii) is inactive (G1269R); iii) shows increased activity (G46E) and iv) shows decreased activity (G50D, R433P, G346R, A439E, R1231H, K1237N).^14^, ^15^

The only disease associated condition related with hAOX1, known so far, is the disorder of the purine metabolism caused by XO and AOX combined deficiency and named type II xanthinuria. Mutation of human Moco sulfurase gene is responsible for classical type II xanthinuria, due to the failure of the mechanism responsible for inserting the essential sulfur atom into the active center of hAOX1 and XO.^16^ The presence of nsSNPs in hAOX1 leading to loss of the Moco insertion may also be related with the presence of type II xanthinuria disease conditions and should be further investigated.

To the best of our knowledge, the only reported pharmacogenetic study related with hAOX1 was that of patients treated with a synthetic derivative of quinoxaline phenoxypropionic acid (XK469), which is a selective topoisomerase II β inhibitor, eliminated mainly via hAOX1 metabolism.^17^ A gene study was performed to investigate whether the genetic variation of hAOX1 contributed to interindividual variability observed in XK469 clearance. The study evaluated whether 41 hAOX1 nsSNPs and seven liver expression quantitative trait loci (eQTLs) were associated with XK469 plasma clearance, but the study was inconclusive and variability in XK469 clearance could not be attributed to polymorphisms in the AOX1 gene.

In this work, we utilized a collection of computational tools with concordance analysis approaches to predict putative phenotypic effects—deleterious or nondeleterious—and protein stability changes—stabilizing, destabilizing, or neutral—of all validated hAOX1 nsSNPs deposited in the dbSNP. The corresponding amino acid substitutions were grouped according to their location in the crystal structure, and possible structural implications were also discussed. This study constitutes the first extensive analysis for the presence of nsSNPs in hAOX1 and can be used to guide future pharmacogenetic, structural and functional studies regarding interindividual variability of the human enzyme.

## 2 MATERIALS AND METHODS

### 2.1 Identification of nsSNPs

The protein sequence and polymorphism data for hAOX1 were collected from UniProt^18^, ^19^ [http://www.uniprot.org/] and NCBI dbSNP^11^ [http://www.ncbi.nlm.nih.gov/SNP/] databases, respectively. Note that the NCBI dbSNP accepts information on SNPs derived from both experimental and computational methods, corresponding to SNPs with and without validation evidences, respectively. In this study, only the validated nsSNPs were used for analysis. Validation details was obtained from the 1000 Genomes browser, by cluster and by frequency.^11^, ^20^, ^21^

### 2.2 Nonsynonymous nsSNPs analysis

We submitted the protein sequence of hAOX1 to eight bioinformatics tools to predict the functional consequences or putative phenotype effects of the nsSNPs: I‐Mutant 2.0,^22^ PolyPhen 2.0,^23^ nsSNPAnalyzer,^24^ PhD‐SNP,^25^ Panther,^26^ SNPs&GO,^27^ PROVEAN,^28^ and SIFT.^29^ We classified the nsSNPs as deleterious or nondeleterious by comparing the results obtained from all programs and when concordance was obtained for at least six of eight programs used. The prediction accuracy was improved by performing the concordance analysis of nsSNPs using the tools mentioned above.

### 2.3 Protein stability analysis

To evaluate the nsSNP‐induced changes on protein stability, we submitted the sequence and structure of hAOX1 to the following web servers: I‐Mutant 3.0 (sequence or structure‐based),^30^ INPS (sequence or structure‐based),^31^ DUET (structure based),^32^ SDM (structure based),^33^ mCSM (structure based),^34^ and MuPRO (sequence and structure‐based).^35^ The nsSNPs were predicted as destabilizing, stabilizing or neutral, in the last case if no effect on the protein structure was predicted, by comparing the values of the free energy change (ΔΔG) obtained by at least four of eight programs.

The datasets used for all predictor programs were obtained from ProTherm, which is a comprehensive collection of thermodynamic parameters for *wt* and mutant proteins database.^36^ It measures the free energy change value (ΔΔG) by computing the unfolding Gibbs free energy (ΔG) for the native form and subtracting it from that of the mutant form. The ΔΔG values are listed in Table S1 in Supplement, but for clarity, only the output of I‐Mutant 3.0^30^ is described in the text, since this uses a structure‐based analysis. The basic methodology and web availability of each nsSNPs functional and stability analysis tools is explained in the supplementary section.

The 3D structure of hAOX1 *wt* was retrieved from Protein Data Bank [www.rcsb.org] (PDB code 4UHW—substrate free form) and the missing regions—particularly the linker 1 region (residues 167‐230), were modeled using the program Modeller.^37^

### 2.4 Localization of the nsSNPs in the crystal structure

All the nsSNPs predicted to be deleterious in at least six of eight different in silico tools used and found to be simultaneously validated in the NCBI‐dbSNP database, were mapped in the crystal structure of hAOX1 using Coot^38^ and PyMol.^39^ Also, the LigPlot program^40^ was used to identify all the residues interacting with the protein cofactors.

## 3 RESULTS

### 3.1 SNPs identification and stability analysis

**Figure 2:**
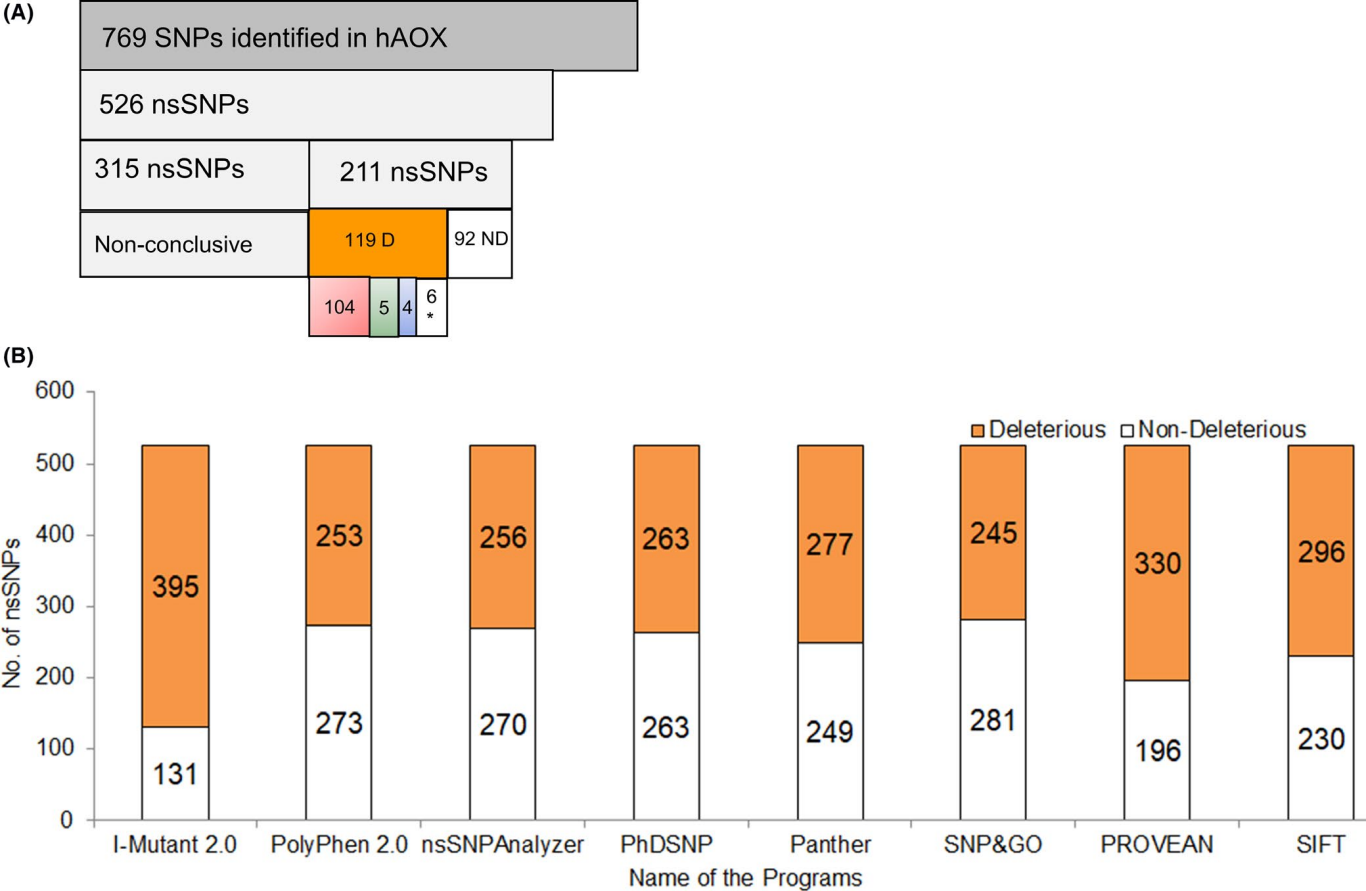
Screening of hAOX1 nsSNPs available at the NCBI‐dbSNP data base using concordance analysis: (A) overall statistics; (B) prediction of putative phenotypic effects of 526 nsSNPs for hAOX1 using 8 programs. The nsSNPs were classified as deleterious (D) or nondeleterious (ND) if concordance in at least 6/8 programs was obtained. * represents nonconclusive

As to date, in the NCBI‐dbSNP database, a total of 769 SNPs was found in hAOX1, from which 526 belong to the nonsynonymous functional category and were further selected for the analysis (Figure 2A). Detailed experimental investigation for understanding the functional effects of all nsSNPs is a time‐consuming and cumbersome process. Bioinformatics tools were therefore used to identify and prioritize the significant and putative deleterious nsSNPs for further experimental studies. Deleterious nsSNPs may be responsible for inducing disease associated phenomena or structural alterations in proteins and their identification is possible through computational work. The accuracy for identifying the deleterious nsSNPs can be increased by combining the results provided by several different bioinformatics tools with a concordance analysis approach.^41^

In this study, eight different computational programs were used to understand the functional consequences or putative phenotypic effects of the 526 nsSNPs. Because each algorithm uses different parameters to identify nsSNPs, only the ones considered as deleterious in at least six of the eight programs were selected for further analysis. By comparing the results obtained from the prediction tools, 119 nsSNPs were found to be deleterious and 92 nondeleterious (Figure 2A and B). The prediction results of remaining 315 nsSNPs are nonconclusive and hence they were excluded for further stability analysis. All the 119 deleterious variants are described in Table S1, including the Minor Allele Frequency (MAF) details and predicted ΔΔG values from all the programs.

To predict the protein stability‐changes induced by the presence of polymorphism in the 119 putative deleterious nsSNPs, we used a series of sequence and structure‐based stability prediction programs (six programs, eight outputs in total), as detailed in the Materials and Methods and Supplementary section. Stability analysis results showed that, out of the 119 deleterious variants, 104 might be responsible for destabilizing the protein structure. In contrast, five nsSNPs are predicted to have a stabilizing effect on the protein structure, namely T53I, H100L, Q776V, T1053I, and S1271L. Furthermore, G48V, H340Y, R906W, and S1194N variants do not seem to have any effect on protein structure stability and are considered neutral. Finally, the prediction was inconclusive for Q348R, G452R, G482E, T594M, T706I, and A1167V nsSNPs.

### 3.2 Structural localization of nsSNPs in hAOX1

**Figure 3:**
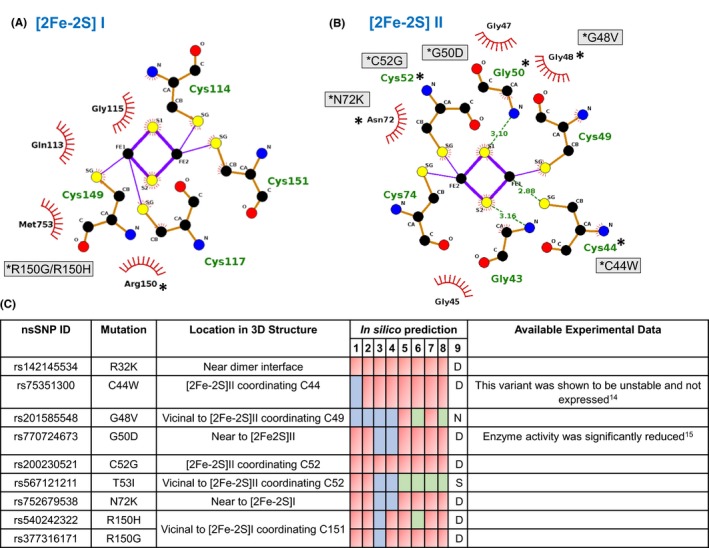
Location of nsSNPs in domain I of hAOX. Close‐up of the (A) [2Fe‐2S] I center (B) [2Fe‐2S] II center, the variant residues are marked with a * and the corresponding nsSNPs represented in grey boxes. (C) In silico prediction results for the nine most relevant nsSNPs using: I‐Mutant (Structure input), 1; I‐Mutant (Sequence input), 2; INPS (Sequence input), 3; INPS (Structure input), 4; SDM (Structure input), 5; mCSM (Structure input), 6; Duet (Structure input), 7; MUpro (Sequence with Structure input), 8; and Consensus prediction, 9. The effect of the variant on the structure stability is depicted using color code: destabilizing variants in red, stabilizing variants in green and neutral variants in blue**.** Experimental data were obtained with the purified recombinant enzyme using a codon‐optimized construct, with the assumption that the enzyme efficiency in vivo shall be comparable

**Figure 4:**
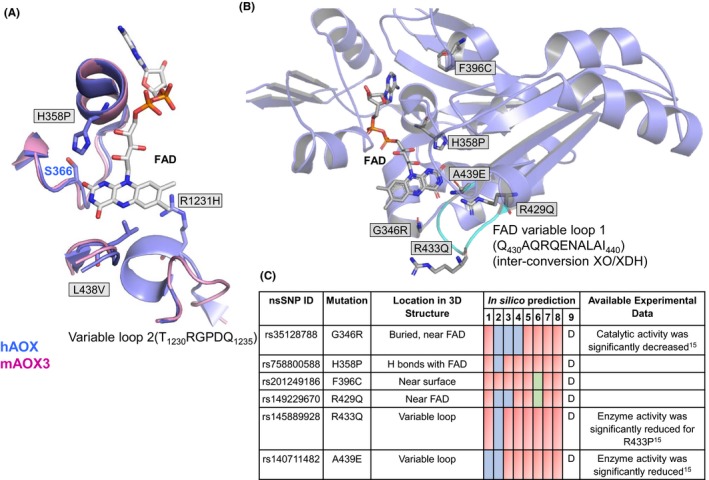
(A) Superposition of hAOX1 (PDB ID: 4UHW; blue) and mAOX3 (PDB ID: 3ZYV; pink) crystal structures at the FAD binding site (solvent view point). The FAD is represented color‐coded and correspond to the hAOX1 crystal structure. (B) Location of nsSNPs in domain II of hAOX1. (C) Stability prediction results of most relevant nsSNPs in domain II using: I‐Mutant (Structure input), 1; I‐Mutant (Sequence input), 2; INPS (Sequence input), 3; INPS (Structure input), 4; SDM (Structure input), 5; mCSM (Structure input), 6; Duet (Structure input), 7; MUpro (Sequence with Structure input), 8; and Consensus prediction, 9. The effect of the variant on the structure stability is depicted using color code: destabilizing variants in red, stabilizing variants in green, and neutral variants in blue. Experimental data were obtained with the purified recombinant enzyme using a codon‐optimized construct, with the assumption that the enzyme efficiency in vivo shall be comparable

**Figure 5:**
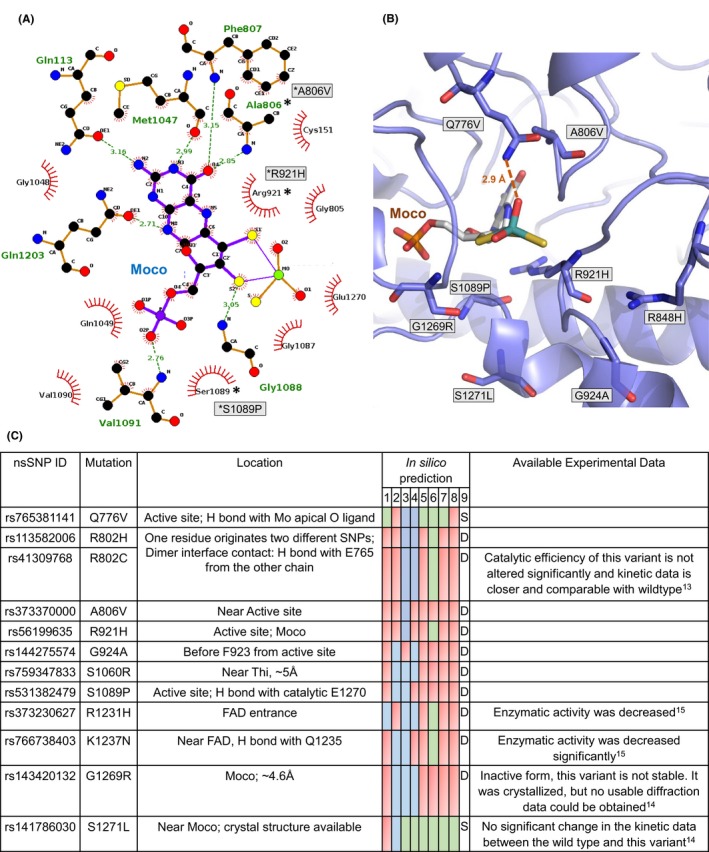
Location of nsSNPs in Domain III of hAOX1 (A) Two dimensional representation of Moco in hAOX1 prepared by Ligplot.^40^ The variant residues are marked with a * and the corresponding nsSNPs represented in grey boxes; (B) Representation of the Mo active site and surrounding residues in hAOX1, (C) In silico stability prediction results of nsSNPs in Domain III. Experimental data were obtained with the purified recombinant enzyme using a codon‐optimized construct, with the assumption that the enzyme efficiency in vivo shall be comparable

In this study, of 119 putative deleterious nsSNPs, we analyzed only the 37 nsSNPs according to their location in the different domains (Figure 1). The remaining nsSNPs, located at the protein surface, are listed in Table S1. Figure 1 depicts the approximate localization of some of the deleterious variants in the different domains and linker region of the hAOX1 3D structure as well as on the dimer interface. In the following sections, and whenever available, the predicted stabilities are compared with published experimental data on catalytic efficiencies, obtained for recombinant hAOX1 variants (Figures 3C, 4C, 5C).^13^, ^14^, ^15^

#### 3.2.1 nsSNPs at the hAOX1 domain I

The hAOX1 domain I comprises two [2Fe‐2S] centers and the linker1 region (Figure 1). Our results show that there are 19 nsSNPs in domain I, corresponding to 16% of the total number of predicted deleterious variants (Table S1). These nsSNPs are located either at the surface of the protein or close/within the [2Fe‐2S] coordinating motifs: 16 variants are classified as destabilizing; 2 as stabilizing; and 1 as neutral.

Two of these nsSNPs correspond to Cys residues coordinating the Fe atoms from [2Fe‐2S] II, C44W and C52G (Figure 3)A and B). Our bioinformatics analysis suggested that the C44W and C52G (prediction supported by 7/8 and 8/8 programs, respectively) are deleterious variants that affect the protein stability, with ΔΔG values of −0.26 and −1.22 kcal/mol, respectively (Figure 3C and Table S1). This is in agreement with previous experimental results where hAOX1‐C44W variant could not be produced by heterologous expression.^14^ The inability to synthesize a stable C44W protein variant can be explained by the fact that replacing Cys by a Trp residue results in loss of the FeS center incorporation, leading to an unstable form of the protein. Assembly of the FeS clusters is one of the first and crucial steps in protein maturation and its lack may lead to a structurally disordered enzyme, in which the cofactor cannot be inserted.^42^

In domain I, we can also find nsSNPs vicinal to the FeS coordinating cysteines (G48V, G50D, T53I, N72K, and R150H/G) (Figure 3A and B). The G50D variant is predicted to be deleterious, showing a ΔΔG value of −0.71 kcal/mol, destabilizing the protein structure (6/8 programs) (Table S1 and Figure 3C). This is in agreement with a recent study where this mutant was biochemically characterized showing similar Km values as the wild type, but 20% reduced activity towards oxidation reactions.^15^ The substitution of G50 by a larger, charged residue (Asp) would result in destabilization of the neighboring residues, with direct impact on Cys49 residue, which is coordinating [2Fe‐2S] II. The same study includes G46E variant, which also shows a similar Km but a 2‐fold increase in *k*_cat_, suggesting that this variant does not have a negative effect on the protein.^15^

Foti and coauthors suggested in 2017 that G46E gives rise to a variant with increased oxidation activity.^15^ Although the nature of the side chains of Gly and Glu residues are very different, and a large impact in the structure could have been anticipated, our in silico results predict that this nsSNP is nondeleterious, corroborating the experimental data.^15^ The loop that harbors G46 is in the electron path involving [2Fe‐2S] II and the Glu side chain might contribute to a faster electron flow.

The analysis of the N72K variant, also within the [2Fe‐2S] II coordination sphere, with a predicted ΔΔG of −0.48 kcal/mol (supported by 6/8 programs), suggests that this variant will probably alter not only the structure, but also the electrostatic potential of the cluster, probably influencing the electron transfer reaction. In this domain, and close to [2Fe‐2S] I, R150 generates two variants—R150H/G. This Arg residue is vicinal to the coordinating Cys149, at 8 Å from the pterin cofactor, and responsible for a salt bridge with E1218. Its mutation to a smaller residue (His or Gly) will prevent this type of interaction, probably destabilizing the cofactor‐binding region. The bioinformatics analysis here performed predicts that this mutation will have a deleterious effect, more pronounced for Gly than His residue, as suggested by the ΔΔG values obtained of −0.92 and −0.74 kcal/mol.

#### 3.2.2 nsSNPs at the linker I

Only the G177E nsSNPs was found to be in the linker1 region of the protein. In the hAOX1 crystal structure, this is a mobile region that could not be defined in the electron density map (residues 167‐199) and therefore the structural effect of this nsSNP cannot be anticipated by a simple structural analysis.^43^ However, according to this in silico tools, this nsSNP is deleterious and affects the stability of protein structure with the predicted ΔΔG value of −0.93 kcal/mol (Table S1 in Supplement).

#### 3.2.3 nsSNPs at the hAOX1 domain II

The FAD cofactor in domain II of hAOX1 is responsible for transferring the electrons generated during the catalytic reaction to the terminal electron acceptor, which is molecular oxygen (Figure 1). A total of 21 nsSNPs were found to be located at the FAD domain, which corresponds to 18% of total number of predicted deleterious nsSNPs (Table S1). Of these, 17 variants are predicted to have a destabilizing effect on the protein structure, whereas only one (H340Y) corresponds to a neutral variant (Table S1). The prediction results of other 3 nsSNPs are nonconclusive (Q348R, G452R, and G482E).

Important variants of this domain are H358P and R429Q variants (Figure 4). H358 residue is located in the middle of an α‐helix and establishing H bonds with S366 residue and with two oxygen atoms (O2 and O4) from the FAD moiety. Replacement of this His by Pro residue will most probably disturb the α‐helix regular pattern and led to destabilization of the FAD cofactor. In the structure, R429 residue is opposite to H358, and H bonded to N3 and O4 atoms of the FAD, being involved in a salt bridge with D367 residue. These H358P and R429Q variants probably correspond to nonfunctional forms of hAOX1, and in silico prediction gives a ΔΔG value of −0.60 and −0.92 kcal/mol, respectively.

**Figure 6:**
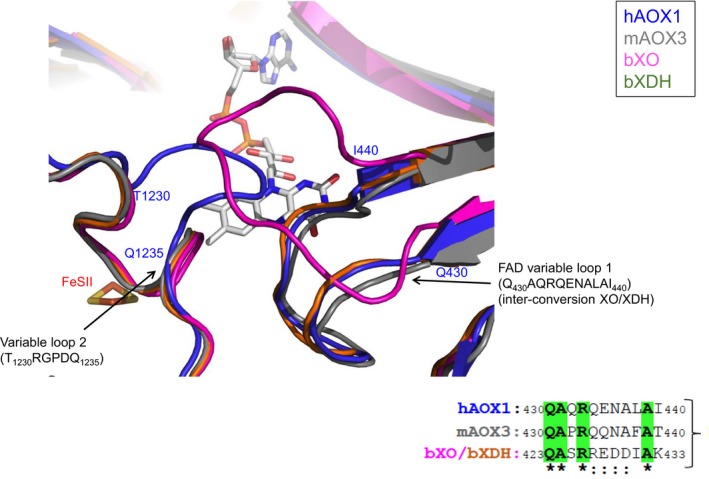
Superposition of hAOX1 (PDB ID: 4UHW; blue), mAOX3 (PDB ID: 3ZYV; gray), bXO (PDB ID: 1FIQ; pink) and bXDH (PDB ID: 3UNI; green) crystal structures at the FAD binding site

Other relevant nsSNPs in this region are R1231H and K1237N, which, although belonging to the Moco domain III, have in the hAOX1‐phthalazine‐thioridazine structure, the respective side chains at 7.2 Å and 6.8 Å from the FAD, respectively. The R1231H variant is located at the entrance of the FAD pocket and is part of a loop region (variable loop 2, T_1230_RGPDQ_1235_) that, in the human enzyme, is flipped almost 180° when compared to the corresponding loop in the mAOX3 and bovine XO structures (Figure 6). The R1231H and K1237N mutations produce much less active forms of the enzyme (with 2 and 3.5 times lower k_cat_ than the *wt* enzyme).^15^ The ΔΔG values here obtained (−0.66 and −1.13 kcal/mol) suggest a structure destabilization for both variants. The crystal structure of R1231H is currently under refinement and shall provide relevant input to better understand the possible implications of this nsSNP.

Two nsSNPs, R433Q and A439E are part of the FAD variable loop 1 (Q_430_AQRQENALAI_440_), which is described in XO as responsible for the XO‐XDH inter‐conversion.^44^ These residues are conserved in mAOX3 and bXO/XDH, and the crystal structure of hAOX1 shows the side chain of R433 residue is H bonded to main chain atoms of the loop. Our in silico analysis suggests that these two variants (R433Q and A439E) will highly destabilize the protein (with ΔΔG values of −1.05 and −0.82 kcal/mol, respectively) and experimental data showed that R433P and A439E variants have decreased oxidation activity, with 4 and 5 times lower k_cat_ compared to the *wt* enzyme, respectively.^15^ The recent kinetic^15^ and structural (not published) data concerning the A437V and L438V nsSNPs from the variable loop 1 showed interesting results. Our computational studies predicted that these correspond to nondeleterious nsSNPs, which is in accordance with the steady‐state kinetic results that have shown that the Km values of the variants are comparable to that of the *wt*.^15^ In contrast, the value of k_cat_ resulted in a similar value for A437V, and a 1.2 increase for L438V.^15^ During the hAOX1 catalytic oxidation, reduction of oxygen species occurs, and H_2_O_2_ and O^2−^ are released.^45^, ^46^ The authors observed that a significantly increased rate of superoxide production was obtained for the L438V variant (72%).^15^

In the FAD domain we can also find buried nsSNPs, located close to the FAD and to the [2Fe‐2S] I cluster, that destabilize the protein structure. This is the case of G346R variant, where the introduction of a bulky and charged side chain is predicted to have a deleterious effect (ΔΔG −0.05 kcal/mol). This prediction is in agreement with experimental data on G346R, whose catalytic activity is decreased (also 4 times lower k_cat_ when compared to *wt* enzyme).^15^ F396C, although at the surface of the protein, has its side chain pointing towards the FAD isoalloxazine ring, in a hydrophobic pocket. The destabilizing effect of this variant is predicted to be very pronounced, possibly promoting important conformational changes (with ΔΔG value of −1.42 kcal/mol).

#### 3.2.4 nsSNPs at the hAOX1 domain III

The majority of hAOX1 nsSNPs (65%) are located in the *C*‐terminal region of the Moco domain III, with 71 destabilizing variants and three stabilizing variants (Q776V, T1053I and S1271L). The prediction results of the three variants T594M, T706I, and A1167V in this domain are nonconclusive, while variants R906W and S1194N are predicted as neutral. The detailed results of this prediction are given in Table S1 and Figure 5C.

The nsSNPs near the Moco active site and surrounding region are the most relevant ones for enzyme activity and substrate specificity. Some of these nsSNPs have been expressed and characterized, namely R802C, R921H, G1269R, and S1271L (Table S1 and Figure 5C).^13^, ^14^ Interestingly, in the first report concerning the presence of nsSNPs in hAOX1, it was shown that the R802C nsSNP was predominantly purified in its monomeric form in solution, resulting in a higher proportion of the inactive form of the enzyme.^13^ Nevertheless, the catalytic efficiency of the dimeric portion of the protein was not affected and similar values were obtained in comparison to the *w*t hAOX1. In the hAOX1 crystal structure, residue R802 from chain B establishes a salt bridge with E765 residue from chain A, and this could explain why the R802C variant was predominantly obtained in its monomeric form. In fact, R802 residue gives rise to two variants, R802C and R802H, both predicted to destabilize the protein structure (ΔΔG of −0.58 and −0.79 kcal/mol, respectively), probably because the mutated side chains cannot promote inter‐molecular electrostatic interactions.

In the case of G1269R variant, experimental results have shown that, although metal analysis suggests that all cofactors are present, the enzyme is inactive.^14^ Gly1269 residue is located immediately before the catalytically essential Glu1270 and its modification to a large and positively charged residue as Arg, will likely promote large structural changes to allow accommodating the bulky side chain, affecting the interactions between the cofactor and the surrounding residues, hence impacting protein stability and lack of activity (ΔΔG: −0.76 kcal/mol).

The S1271L nsSNP, also adjacent to the catalytically essential Glu1270 residue, was so far identified to be heterozygous in all the tested individuals^13^ and, according to six of eight programs used, is predicted to be stabilizing the protein structure (Table S1 and Figure 5C). Biochemical analysis and kinetic data however, showed that the resulting protein variant has similar characteristics to the *wt* enzyme. Also, the crystal structure of this variant, the first published structure of a hAOX1 nsSNP (PDB ID: 5EPG), showed no major structural deviations.^14^ S1089P is another destabilizing variant, with a predicted ΔΔG value of −0.75 kcal/mol. The hAOX1 crystal structure shows that Ser side chain is hydrogen bonded to Glu1270 residue and is part of the loop that surrounds the pyranopterin dithiolene moiety (S1086‐S1089). A Pro residue at this position is likely to disrupt the structure of the loop, and impair the intra‐molecular interactions required for cofactor stabilization.

Also, close to the Mo active site, we find R921H, G924A and A806V variants. R921 side chain is almost parallel to the pterin moiety of Moco (at *ca* 3‐4 Å) and is in disallowed regions of the Ramachandran plots of all XO family members’ crystal structures. The ΔΔG value obtained for this residue (−0.76 kcal/mol), suggests that its substitution by a His residue will possibly prevent cofactor accommodation producing a less active enzyme. In the same loop, we find G924A, also a destabilizing variant (ΔΔG: −0.62 kcal/mol). A806 residue is positioned sideways to the pterin rings, H bonded to O4 atom via its amino group. In silico analysis suggests that its replacement by a larger residue will impact, although to less extent, the stability of the structure (ΔΔG = −0.44 kcal/mol).

Another interesting nsSNP at the active site is Q776V, but so far, no kinetic data are available. Q776 residue is a highly conserved residue among XO and AOX family members,^9^, ^47^, ^48^ that makes an H bond with the Mo apical oxygen ligand (2.9 Å) (Figure 5A and B). The substitution of Gln by a Val residue might influence the catalytic activity of the enzyme but not the structure of the protein, and according to our in silico results, this nucleotide change gives rise to a stabilizing nsSNP, but with no relevant effect on the protein structure (ΔΔG = 0 kcal/mol).

Domain III includes also the noncompetitive inhibition site, where thioridazine, and likely other phenothiazine family members, binds. One of the loops at the surface of the protein that accommodates the inhibitor contains S1060 residue and its variant S1060R is predicted as deleterious and structure destabilizing variant. Showing a high ΔΔG value (−0.94 kcal/mol), this variant's side chain will very likely unstructure the inhibitor pocket and decrease the binding affinity, influencing the inhibitory effect of this family of molecules.

Several nsSNPs were found to involve the same residue, namely: G741D/S, G746V/R, R802H/C, L914R/F, A1028P/V, R1109C/H, and E1170K/R (Table S1). Most of them are located in the Moco domain. This might be related with the mechanisms of mutation induction, and no further evidence could be found to explain this occurrence.

#### 3.2.5 nsSNPs at the dimerization interface

Several nsSNPs are found at the dimerization interface or very close to it: R32K, G595E, P762L, A1026T, A1028P/V, Y1033C, A1102D, and R1109C/H (Figure 1 and Table S1). All these variants are predicted to be deleterious and destabilizing the structure of the protein, showing high ΔΔG values. It is worth mentioning R32K: in the crystal structure of hAOX1 its side chain while pointing towards the protein interior, is involved in a salt bridge with D601 and D602 residues of the same monomer and 8/8 programs suggest that its mutation to a Lys residue will have a deleterious effect. Although this variant corresponds to a mutation with an equally charged residue, its side chain is slightly shorter and with a higher degree of flexibility, which might be enough to diminish electrostatic interactions, unstructuring this region, and destabilizing the protein (Figure 3C and Table S1 in Supplement). N1135S variant, also located at the dimerization interface, was classified by Hartman et al as a fast metabolizer, producing a modest increase in the catalytic efficiency of the variant, although this effect was not observed across all tested substrates.^13^ N1135 establishes two H bonds with main chain atoms of S1137 and one with side chain atom of R1109 residue, but it is not involved in major inter‐molecular interactions*.* In agreement, our in silico analysis suggests that this nsSNP is nondeleterious, with no impact on the protein stability.

## 4 DISCUSSION AND CONCLUSIONS

All residues located at the active site, near the protein cofactors, noncompetitive inhibition site, or in the electron transfer pathway, have a higher probability of affecting the enzyme activity or disrupting the protein secondary and/or tertiary structure, when replaced by a different residue. On the contrary, modification of residues located at the protein surface should cause no major impact on the overall structure, catalytic activity or substrate specificity of the enzyme. Several amino acid residues from hAOX1 have been identified as important for its putative biological function. In particular those located at the protein Mo active site, near the [2Fe‐2S] centers and FAD cofactor, as well as in the electron transfer path, also important for the enzyme's catalytic activity.^43^ Our systematic bioinformatics analysis reported 119 deleterious and 92 nondeleterious nsSNPs in hAOX1. Due to the very few studies on this enzyme, the MAF of 119 nsSNPs is less than 1% in the population. However, the obtained in silico data with reference to putative phenotypic and protein structure stability effects may suggest and led to further studies on site directed mutagenesis, biophysical, X‐ray crystallographic and pharmacogenetics characterization.

Consensus structure stability analysis results suggested that 4 variants do not have any impact on hAOX1 while 104 variants (out of 119) might be responsible for destabilizing the protein structure—in some cases inactivating the enzyme—whereas five variants enhance the stability. The prediction results of the remaining six nsSNPs are nonconclusive, requiring further studies. We inspected all 119 putative deleterious nsSNPs in the hAOX1 crystal structure and observed that most nsSNPs are located in domain III—the catalytic domain (90 kDa)—responsible for the substrate oxidation activity and enclosing the Mo active site. From their structural location, we considered only 37 nsSNPs for a more detailed analysis. From the crystal structure analysis, 8 nsSNPs are found in direct or close contact with the [2Fe‐2S] centers, 6 nsSNPs are found near the FAD cofactor and 12 nsSNPs are found at the Moco active site. Moreover, only one nsSNP is identified in linker 1 region (G177E) while 10 nsSNPs are located at the dimer interface. These are the most relevant nsSNPs that deserve further investigation.

The reported results provide new insights regarding the structural and functional impact of point mutations in hAOX1 secondary structure, overall fold and subunit interactions. They also provide hints for predicting the putative effect of the destabilizing nsSNPs upon cofactors stabilization, electron transfer pathway and substrate/inhibitor binding, and correlate with available, although scarce, biochemical and pharmacogenetic data.

To our knowledge, this study constitutes the first extensive analysis for the presence of nsSNPs in hAOX1. We believe that this preliminary investigation provides a systematic route for identification and prioritization of potentially important nsSNPs in hAOX1. It may thus be used, in combination with experimental screening, to select most promising destabilizing variants associated with hereditary disorder related to AOX or XO family members. This investigation will also facilitate future studies on pharmacogenomics and personalized medicine.

## DISCLOSURE

The authors declare no conflict of interest.

## AUTHORS CONTRIBUTIONS

Prof. Maria João Romão and Prof. Teresa Santos‐Silva supervised and conceived the study, participating in the analysis and interpretations, wrote and edited the manuscript. Dr. Jayaraman Muthukumaran and Dr. Catarina Coelho participated the data collection, data analysis, interpretations of finding and drafting the manuscript. All authors read and approved the final version of manuscript.

## References

[1] Gray IC . Single nucleotide polymorphisms as tools in human genetics. Hum Mol Genet. 2000;9:2403‐2408. 10.1093/hmg/9.16.2403 11005795

[2] Syvänen AC . Accessing genetic variation: genotyping single nucleotide polymorphisms. Nat Rev Genet. 2001;2:930‐942. 10.1038/35103535 11733746

[3] Ramensky V , Bork P , Sunyaev S . Human non‐synonymous SNPs: server and survey. Nucleic Acids Res. 2002;30:3894‐3900. 12202775 10.1093/nar/gkf493 PMC137415

[4] Zhou SF , Liu JP , Chowbay B . Polymorphism of human cytochrome P450 enzymes and its clinical impact. Drug Metab Rev. 2009;41:89‐295. 10.1080/03602530902843483 19514967

[5] Romão MJ , Coelho C , Santos‐Silva T , ,et al. Structural basis for the role of mammalian aldehyde oxidases in the metabolism of drugs and xenobiotics. Curr Opin Chem Biol. 2017;37:39‐47. 10.1016/j.cbpa.2017.01.005 28126656

[6] Garattini E , Terao M . The role of aldehyde oxidase in drug metabolism. Expert Opin Drug Metab Toxicol. 2012;8:487‐503. 10.1517/17425255.2012.663352 22335465

[7] Pryde DC , Dalvie D , Hu Q , Jones P , Obach RS , Tran TD . Aldehyde oxidase: an enzyme of emerging importance in drug discovery. J Med Chem. 2010;53:8441‐8460. 10.1021/jm100888d 20853847

[8] Beedham C . The role of non‐P450 enzymes in drug oxidation. Pharm World Sci. 1997;19:255‐263. 10.1023/A:1008668913093 9443166

[9] Mota C , Coelho C , Leimkühler S , ,et al. Critical overview on the structure and metabolism of human aldehyde oxidase and its role in pharmacokinetics. Coord Chem Rev. 2018;368:35‐59. 10.1016/j.ccr.2018.04.006

[10] Sugihara K , Kitamura S , Tatsumi K , Asahara T , Dohi K . Differences in aldehyde oxidase activity in cytosolic preparations of human and monkey liver. Biochem Mol Biol Int. 1997;41:1153‐1160. 9161710 10.1080/15216549700202241

[11] Sherry ST . dbSNP: the NCBI database of genetic variation. Nucleic Acids Res. 2001;29:308‐311. 10.1093/nar/29.1.308 11125122 PMC29783

[12] Smith MA , Marinaki AM , Arenas M , ,et al. Novel pharmacogenetic markers for treatment outcome in azathioprine‐treated inflammatory bowel disease. Aliment Pharmacol Ther. 2009;30:375‐384. 10.1111/j.1365-2036.2009.04057.x 19500084

[13] Hartmann T , Terao M , Garattini E , ,et al. The impact of single nucleotide polymorphisms on human aldehyde oxidase. Drug Metab Dispos. 2012;40:856‐864. 10.1124/dmd.111.043828 22279051 PMC4738704

[14] Foti A , Hartmann T , Coelho C , Santos‐Silva T , Romao MJ , Leimkuhler S . Optimization of the expression of human aldehyde oxidase for investigations of single nucleotide polymorphisms. Drug Metab Dispos. 2016;44:1277‐1285. 10.1124/dmd.115.068395 26842593

[15] Foti A , Dorendorf F , Leimkühler S . A single nucleotide polymorphism causes enhanced radical oxygen species production by human aldehyde oxidase. PLoS ONE. 2017;12:e0182061 10.1371/journal.pone.0182061 28750088 PMC5531472

[16] Ichida K , Matsumura T , Sakuma R , Hosoya T , Nishino T . Mutation of human molybdenum cofactor sulfurase gene is responsible for classical Xanthinuria Type II. Biochem Biophys Res Commun. 2001;282:1194‐1200. 10.1006/bbrc.2001.4719 11302742

[17] Ramírez J , Kim TW , Liu W , ,et al. A pharmacogenetic study of aldehyde oxidase I in patients treated with XK469. Pharmacogenet Genomics. 2014;24:129‐132. 10.1097/FPC.0000000000000023 24300566 PMC3901533

[18] Bateman A , Martin MJ , O’Donovan C , et al. UniProt: the universal protein knowledgebase. Nucleic Acids Res. 2017;45(D1):D158‐D169. 10.1093/nar/gkw1099 27899622 PMC5210571

[19] Apweiler R . The universal protein resource (UniProt) in 2010. Nucleic Acids Res. 2009;38(database issue):D142–D148. 10.1093/nar/gkp846 19843607 PMC2808944

[20] Smigielski EM . dbSNP: a database of single nucleotide polymorphisms. Nucleic Acids Res. 2000;28:352‐355. 10.1093/nar/28.1.352 10592272 PMC102496

[21] Sherry ST , Ward M , Sirotkin K . dbSNP ‐ database for single nucleotide polymorphisms and other classes of minor genetic variation. Genome Res. 1999;9:677‐679. 10.1101/gr.9.8.677 10447503

[22] Capriotti E , Fariselli P , Casadio R . I‐Mutant2.0: predicting stability changes upon mutation from the protein sequence or structure. Nucleic Acids Res. 2005;33(web server):W306–W310. 10.1093/nar/gki375 15980478 PMC1160136

[23] Adzhubei IA , Schmidt S , Peshkin L , ,et al. A method and server for predicting damaging missense mutations. Nat Methods. 2010;7:248‐249. 10.1038/nmeth0410-248 20354512 PMC2855889

[24] Bao L , Zhou M , Cui Y . nsSNPAnalyzer: identifying disease‐associated nonsynonymous single nucleotide polymorphisms. Nucleic Acids Res. 2005;33(web server):W480–W482. 10.1093/nar/gki372 15980516 PMC1160133

[25] Capriotti E , Calabrese R , Casadio R . Predicting the insurgence of human genetic diseases associated to single point protein mutations with support vector machines and evolutionary information. Bioinformatics. 2006;22:2729‐2734. 10.1093/bioinformatics/btl423 16895930

[26] Mi H , Poudel S , Muruganujan A , Casagrande JT , Thomas PD . PANTHER version 10: expanded protein families and functions, and analysis tools. Nucleic Acids Res. 2016;44(D1):D336‐D342. 10.1093/nar/gkv1194 26578592 PMC4702852

[27] Capriotti E , Calabrese R , Fariselli P , Martelli P , Altman RB , Casadio R . WS‐SNPs&GO: a web server for predicting the deleterious effect of human protein variants using functional annotation. BMC Genom. 2013;14(suppl 3):S6 10.1186/1471-2164-14-S3-S6 PMC3665478 23819482

[28] Choi Y , Sims GE , Murphy S , Miller JR , Chan AP . Predicting the functional effect of amino acid substitutions and indels. PLoS ONE. 2012;7:e4668 10.1371/journal.pone.0046688 PMC3466303 23056405

[29] Ng PC , Henikoff S . Predicting deleterious amino acid substitutions. Genome Res. 2001;11:863‐874. 10.1101/gr.176601 11337480 PMC311071

[30] Capriotti E , Fariselli P , Rossi I , Casadio R . A three‐state prediction of single point mutations on protein stability changes. BMC Bioinform. 2008;9(suppl 2):S2‐S6. 10.1186/1471-2105-9-S2-S6 PMC2323669 18387208

[31] Fariselli P , Martelli PL , Savojardo C , Casadio R . INPS: predicting the impact of non‐synonymous variations on protein stability from sequence. Bioinformatics. 2015;31:2816‐2821. 10.1093/bioinformatics/btv291 25957347

[32] Pires DEV , Ascher DB , Blundell TL . DUET: a server for predicting effects of mutations on protein stability using an integrated computational approach. Nucleic Acids Res. 2014;42(W1):W314–W319. 10.1093/nar/gku411 24829462 PMC4086143

[33] Worth CL , Preissner R , Blundell TL . SDM–a server for predicting effects of mutations on protein stability and malfunction. Nucleic Acids Res. 2011;39(web server issue):W215–W222. 10.1093/nar/gkr363 21593128 PMC3125769

[34] Pires DEV , Ascher DB , Blundell TL . MCSM: predicting the effects of mutations in proteins using graph‐based signatures. Bioinformatics. 2014;30:335‐342. 10.1093/bioinformatics/btt691 24281696 PMC3904523

[35] Cheng J , Randall A , Baldi P . Prediction of protein stability changes for single‐site mutations using support vector machines. Proteins. 2006;62:1125‐1132. 10.1002/prot.20810 16372356

[36] Bava KA . ProTherm, version 4.0: thermodynamic database for proteins and mutants. Nucleic Acids Res. 2004;32:D120–D121. 10.1093/nar/gkh082 14681373 PMC308816

[37] Webb B , Sali A . Comparative protein structure modeling Using MODELLER. Curr Protoc Protein Sci. 2016;86:2.9.1‐2.9.37. 10.1002/cpps.20 27801516

[38] Emsley P , Lohkamp B , Scott WG , Cowtan K . Features and development of Coot. Acta Crystallogr Sect D Biol Crystallogr. 2010;66:486‐501. 10.1107/S0907444910007493 20383002 PMC2852313

[39] DeLano WL .The PyMOL Molecular Graphics System. Schrödinger LLC wwwpymolorg. 2002; Version 1.:http://www.pymol.org. citeulike-article-id:240061

[40] Laskowski RA , Swindells MB . LigPlot+: multiple ligand‐protein interaction diagrams for drug discovery. J Chem Inf Model. 2011;51:2778‐2786. 10.1021/ci200227u 21919503

[41] Ali Mohamoud HS , Manwar Hussain MR , El‐Harouni AA , ,et al. First comprehensive in silico analysis of the functional and structural consequences of SNPs in human GalNAc‐T1 gene. Comput Math Methods Med. 2014;2014:1‐15. 10.1155/2014/904052 PMC3960557 24723968

[42] Iobbi‐Nivol C , Leimkühler S . Molybdenum enzymes, their maturation and molybdenum cofactor biosynthesis in *Escherichia coli* . Biochim Biophys Acta ‐ Bioenerg. 2013;1827:1086‐1101. 10.1016/j.bbabio.2012.11.007 23201473

[43] Coelho C , Foti A , Hartmann T , Santos‐Silva T , Leimkühler S , Romão MJ . Structural insights into xenobiotic and inhibitor binding to human aldehyde oxidase. Nat Chem Biol. 2015;11:779‐783. 10.1038/nchembio.1895 26322824

[44] Enroth C , Eger BT , Okamoto K , Nishino T , Nishino T , Pai EF . Crystal structures of bovine milk xanthine dehydrogenase and xanthine oxidase: structure‐based mechanism of conversion. Proc Natl Acad Sci USA. 2000;97:10723‐10728. 10.1073/pnas.97.20.10723 11005854 PMC27090

[45] Kundu TK , Hille R , Velayutham M , Zweier JL . Characterization of superoxide production from aldehyde oxidase: An important source of oxidants in biological tissues. Arch Biochem Biophys. 2007;460:113‐121. 10.1016/j.abb.2006.12.032 17353002 PMC4073616

[46] Kundu TK , Velayutham M , Zweier JL . Aldehyde oxidase functions as a superoxide generating NADH oxidase: an important redox regulated pathway of cellular oxygen radical formation. Biochemistry. 2012;51:2930‐2939. 10.1021/bi3000879 22404107 PMC3954720

[47] Cerqueira NMFSA , Coelho C , Brás NF , ,et al. Insights into the structural determinants of substrate specificity and activity in mouse aldehyde oxidases. J Biol Inorg Chem. 2015;20:209‐217. 10.1007/s00775-014-1198-2 25287365

[48] Mahro M , Brás NF , Cerqueira NMFSA , ,et al. Identification of crucial amino acids in mouse aldehyde oxidase 3 that determine substrate specificity. PLoS ONE. 2013;8:e82285 10.1371/journal.pone.0082285 24358164 PMC3864932

