# Supplementary figures and images for: Systematic exploration of predicted destabilizing nonsynonymous single nucleotide polymorphisms (nsSNPs) of human aldehyde oxidase: A Bio‐informatics study
Source: Pharmacol Res Perspect. 2019 Nov 22;7(6):e00538. doi: 10.1002/prp2.538 (PMC6874515; doi:10.1002/prp2.538)

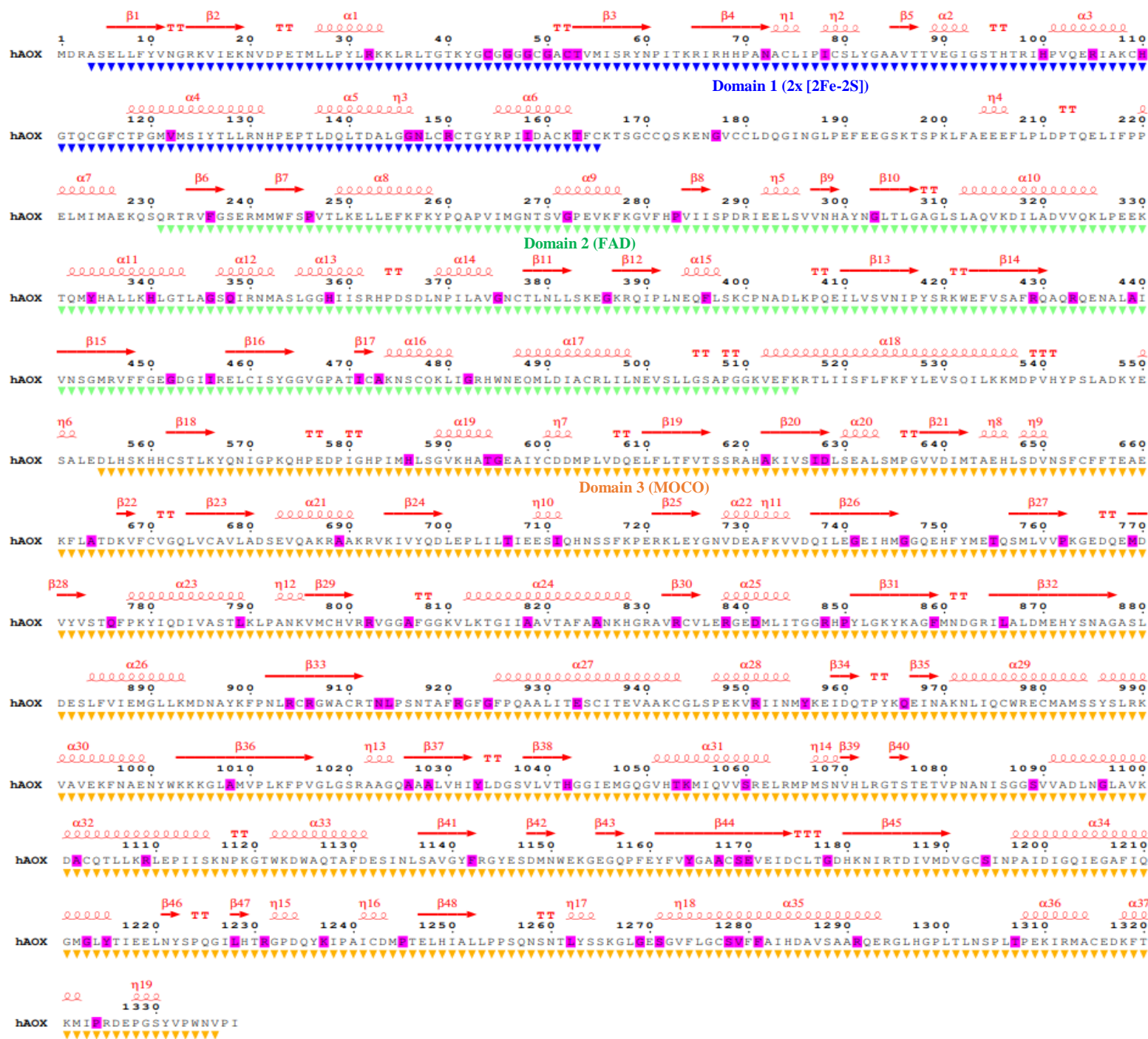

FIGURE S1

Supplement: Supplementary file 1 [file PRP2-7-e00538-s001.pdf]
