# Supplementary material for: Systematic exploration of predicted destabilizing nonsynonymous single nucleotide polymorphisms (nsSNPs) of human aldehyde oxidase: A Bio‐informatics study
Source: Pharmacol Res Perspect. 2019 Nov 22;7(6):e00538. doi: 10.1002/prp2.538 (PMC6874515; doi:10.1002/prp2.538)
Supplement: Supplementary file 2 [file PRP2-7-e00538-s002.doc]

**Materials and Methods:**

**Non-synonymous nsSNPs analysis:**

I-Mutant 2.0 (http://folding.biofold.org/i-mutant/i-mutant2.0.html) is Support Vector Machine (SVM) based predictor which classifies the nsSNPs into deleterious or non-deleterious from sequence information. PolyPhen 2.0 (http://genetics.bwh.harvard.edu/pph2/) predicts possible functional impact of an amino acid substitution using straightforward physical and comparative considerations. nsSNPAnalyzer (http://snpanalyzer.uthsc.edu/) classifies the nsSNPs into deleterious or non-deleterious based on Random forest machine learning method. PhD-SNP (http://snps.biofold.org/phd-snp/phd-snp.html) is also an SVM based predictor which utilizes sequence and profile information to classify the nsSNPs into deleterious or non-deleterious. PANTHER (Protein Analysis Through Evolutionary Relationships) (http://www.pantherdb.org/tools/csnpScoreForm.jsp) classifies nsSNPs into deleterious or non-deleterious based on evolutionary sequence relationship. SNPs&GO (http://snps.biofold.org/snps-and-go/snps-and-go.html) uses functional information codified by gene ontology (GO) terms which classifies the nsSNPs based on SVM machine learning method. PROVEAN (http://provean.jcvi.org/seq_submit.php) predicts the functional impact of single amino acid substitution as well as other classes of sequence variations which include insertions, deletions, and multiple amino acid substitutions based on sequence homology-based approaches. SIFT (https://sift.bii.a-star.edu.sg/www/SIFT_seq_submit2.html) is another computational tool to classify nsSNPs into deleterious or non-deleterious based on sequence similarity search with related sequences followed by multiple sequence alignment, identify the conservation and calculating normalized probabilities of all possible amino acid substitutions from the alignment.

**Protein stability analysis:**

I-Mutant 3.0 (http://gpcr2.biocomp.unibo.it/cgi/predictors/I-Mutant3.0/I-Mutant3.0.cgi) is an SVM based three state predictor using sequence or structural information to classify the nsSNPs into destabilizing mutations, stabilizing mutations and neutral mutations. INPS (https://inpsmd.biocomp.unibo.it/inpsSuite/default/index) extracted descriptor information from input amino acid sequence or three-dimensional structure of protein followed by mapping of these descriptors to ΔΔG values using a support vector regression with a radial basis function kernel. DUET (http://biosig.unimelb.edu.au/duet/stability) uses the two separate approaches namely mCSM (mutation cut-off scanning matrix; predictive model using graph-based signature to represent three-dimensional protein environment) and SDM (site directed mutator; statistical potential energy function) followed by consensus predictions and these were optimized by SVM machine learning method and ΔΔG values of given nsSNPs were computed. MuPro (http://mupro.proteomics.ics.uci.edu/) accepts both protein sequence and/or structure which uses two machine learning approaches (SVM and Neural Network) to predict the effect of single amino acid substitution on protein stability.

**TABLE S1** Minor allele frequency (MAF) and predicted ΔΔG values (kcal/mol) from various sequence and structure based *in silico* programs for 119 deleterious variants for Human Aldehyde Oxidase (**D** = Destabilize the protein structure, **S** = Stabilize the protein structure, **N** = Neutral effects and NC = Non-conclusive)

| **nsSNP ID** | **Exon** | **Position** | **MAF** | **MAF**  **(in %)** | **Domain** | **I-Mutant**  **(Structure)** | **I-Mutant**  **(Sequence)** | **INPS**  **(Sequence)** | **INPS**  **(Structure)** | **DUET**  **(Structure)** | **SDM**  **(Structure)** | **mCSM**  **(Structure)** | **MuPRO**  **(Sequence & Structure)** | **> = 4/8 Prediction** |
| --- | --- | --- | --- | --- | --- | --- | --- | --- | --- | --- | --- | --- | --- | --- |
| rs142145534 | 2 | R32K | A=0.0002/1  (1000 genome); | < 1% (A) | I (FeS) | -1.11 | -0.55 | -1.5 | -1.5 | -1.17 | -2.1 | -1.39 | -1.32 | **D** |
| rs75351300 | 3 | C44W | Not given | Not given | I | -0.26 | 0.16 | -2.13 | -2.09 | -1.38 | -1.91 | -1.14 | -1.22 | **D** |
| rs201585548 | 3 | G48V | T=0.0002/1 (1000Genomes) | < 1% (T) | I | -0.08 | 0.02 | 0.13 | -0.37 | -0.24 | 0.99 | -0.48 | 0.03 | **N** |
| rs770724673 | 3 | G50D | Not given | Not given | I | -0.71 | -0.66 | -0.75 | -0.92 | -2.76 | -2.89 | -2.44 | 0.004 | **D** |
| rs200230521 | 3 | C52G | G=0.0002/1 (1000Genomes) | < 1% (G) | I | -1.22 | -1.26 | -3.02 | -3.31 | -0.28 | -3.65 | -0.03 | -2.04 | **D** |
| rs567121211 | 3 | T53I | T=0.0002/1 (1000Genomes) | < 1% (T) | I | -0.33 | -0.32 | -0.56 | -0.89 | 1.31 | 2.88 | 1.09 | 0.06 | **S** |
| rs752679538 | 4 | N72K | Not given | Not given | I | -0.48 | -0.12 | -0.29 | -0.66 | -0.05 | -1.31 | -0.04 | -1.15 | **D** |
| rs201859570 | 4 | I78T | C=0.0008/4 (1000Genomes) | < 1% (C) | I | -2.37 | -1.99 | -1.73 | -2.08 | -2.86 | -4.03 | -2.7 | -1.78 | **D** |
| rs564342009 | 4 | H100L | T=0.0002/1 (1000Genomes) | < 1% (T) | I | -0.15 | 0.58 | 0.54 | 0.26 | -0.67 | 2.24 | -0.65 | 0.11 | **S** |
| rs771547350 | 5 | R105K | Not given | Not given | I | -1.09 | -0.37 | -0.95 | -1.39 | -1.31 | -1.33 | -1.16 | -1.03 | **D** |
| rs760326411 | 5 | H110R | G=0.0001/2 (GnomAD) | < 1% (G) | I | -0.5 | 0.10 | -0.53 | -0.70 | -1.5 | -2.32 | -1.36 | -0.34 | **D** |
| rs369685838 | 5 | V122M | A=0.0001/1 (GoESP) | < 1% (A) | I | -0.68 | -1.14 | -0.54 | -1.10 | -1.11 | -0.08 | -1.03 | -0.53 | **D** |
| rs144603555 | 5 | G146S | A=0.0002/1 (1000Genomes) | < 1% (A) | I | -0.33 | -1.14 | -0.73 | -0.79 | -0.8 | 0.36 | -0.93 | -0.2 | **D** |
| rs762784565 | 6 | N147S | Not given | Not given | I | -0.64 | -0.44 | -0.14 | -0.59 | -0.09 | -0.88 | -0.16 | -1.16 | **D** |
| rs540242322 | 6 | R150H | A=0.0002/1 (1000Genomes) | < 1% (A) | I | -0.74 | -1.12 | -0.75 | -1.07 | -1.96 | 0.47 | -1.72 | -0.83 | **D** |
| rs377316171 | 6 | R150G | T=0.0001/1 (GoESP) | < 1% (T) | I | -0.92 | -1.38 | -0.2 | -1.38 | -2.54 | -5.68 | -1.89 | -1.19 | **D** |
| rs754234851 | 6 | I158T | Not given | Not given | I | -1.67 | -1.98 | -2.26 | -2.57 | -3.06 | -4.03 | -2.87 | -2.01 | **D** |
| rs780567839 | 6 | T163P | Not given | Not given | I | -1.09 | -0.62 | -1.11 | -1.48 | -0.6 | -1.74 | -0.39 | -1.31 | **D** |
| rs542431016 | 7 | G177E | A=0.0002/1 (1000Genomes) | < 1% (A) | Linker1 | -0.93 | -0.55 | 0.3 | -0.29 | -0.47 | 2.41 | -0.67 | -0.59 | **D** |
| rs570720452 | 9 | F236L | C=0.0002/1 (1000Genomes) | < 1% (C) | II (FAD) | -1.37 | -0.75 | -0.37 | -1.36 | -2.03 | -0.55 | -1.88 | -0.66 | **D** |
| rs538141326 | 9 | P246S | T=0.0002/1 (1000Genomes) | < 1% (T) | II | -2.07 | -1.33 | -1.07 | -1.11 | -2.78 | 0.03 | -2.83 | -0.77 | **D** |
| rs747444891 | 10 | G272V | Not given | Not given | II | -0.21 | -0.55 | 0.48 | -0.19 | -0.34 | 0.99 | -0.44 | -0.17 | **D** |
| rs766081863 | 10 | P283L | Not given | Not given | II | -0.21 | -0.52 | -0.92 | -0.75 | -0.46 | 0.67 | -0.65 | 0.007 | **D** |
| rs776588487 | 11 | G303E | A=0.0001/20 (GnomAD_exomes) | < 1% (A) | II | -1.03 | -0.51 | -0.29 | -0.73 | -2.5 | -1.3 | -2.36 | -0.6 | **D** |
| rs765471606 | 11 | Y334S | Not given | Not given | II | -2.02 | -0.86 | -2.09 | -2.66 | -4.09 | -3.9 | -4.03 | -1.32 | **D** |
| rs756440136 | 11 | H340Y | Not given | Not given | II | -0.28 | 0.49 | -0.06 | -0.06 | 0.62 | 0.62 | 0.5 | -0.26 | **N** |
| rs35128788 | 11 | G346R | Not given | Not given | II | -0.05 | -0.37 | -0.06 | -0.50 | -0.87 | -3.71 | -0.75 | -0.61 | **D** |
| rs775029057 | 11 | Q348R | Not given | Not given | II | -0.32 | -0.19 | -0.3 | -0.55 | 0.31 | -1.49 | 0.3 | -0.2 | NC |
| rs758800588 | 12 | H358P | Not given | Not given | II | -0.6 | -0.14 | -1.13 | -1.72 | -0.8 | -2.9 | -0.36 | -0.99 | **D** |
| rs761848463 | 12 | G375D | A=0.0001/20 (GnomAD_exomes) | < 1% (A) | II | -1.49 | -0.95 | -0.62 | -1.08 | -1.8 | -0.44 | -1.73 | -0.37 | **D** |
| rs773300881 | 13 | G386R | Not given | Not given | II | -0.65 | -0.36 | -0.43 | -0.30 | -0.02 | 1.35 | -0.44 | -0.68 | **D** |
| rs201249186 | 13 | F396C | G=0.0002/2 (GoESP) | < 1% (G) | II | -1.42 | -1.56 | -2.48 | -2.93 | -2.42 | 0.72 | -2.5 | -0.72 | **D** |
| rs149229670 | 14 | R429Q | A=0.0004/2 (1000Genomes) | < 1% (A) | II | -0.92 | -0.62 | -0.58 | -1.16 | -1.05 | 0.45 | -1.03 | -0.58 | **D** |
| rs145889928 | 14 | R433Q | A=0.0002/2 (GoESP) | < 1% (A) | II | -1.05 | -0.75 | -1.29 | -1.29 | -0.89 | -1.65 | -0.63 | -0.37 | **D** |
| rs140711482 | 14 | A439E | A=0.0001/17 (GnomAD_exomes) | < 1% (A) | II | -0.82 | -0.64 | -1.23 | -1.35 | -1.56 | -2.61 | -1.51 | -0.82 | **D** |
| rs768934046 | 14 | G452R | Not given | Not given | II | -0.48 | -0.52 | 0.64 | -0.27 | -0.52 | -2.77 | -0.64 | -0.62 | NC |
| rs748570268 | 9 | I456S | Not given | Not given | II | -1.9 | -1.83 | -3.28 | -3.35 | -3.61 | -2.93 | -3.46 | -1.43 | **D** |
| rs751391691 | 14 | I471N | Not given | Not given | II | -2.32 | -1.84 | -2.78 | -2.80 | -0.58 | -1.24 | -0.63 | -1.13 | **D** |
| rs72551311 | 14 | A473V | Not given | Not given | II | -0.15 | 0.11 | -0.13 | -0.60 | -0.58 | 0.26 | -0.63 | -0.65 | **D** |
| rs546994774 | 14 | G482E | A=0.0002/1 (1000Genomes) | < 1% (A) | II | -0.75 | -0.63 | -0.8 | -0.86 | -1.25 | -3.46 | -1.19 | -0.69 | NC |
| rs373513974 | 17 | H586D | G=0.0001/1 (GoESP) | < 1% (G) | III (Moco) | -0.61 | -0.21 | -1.08 | -1.54 | -1.1 | -3.12 | -0.74 | -0.72 | **D** |
| rs760108206 | 17 | T594M | T=0.0001/15 (GnomAD_exomes) | < 1% (T) | III | -0.15 | 0.04 | -0.69 | -0.94 | 0.02 | 0.98 | -0.02 | -0.21 | NC |
| rs751152293 | 17 | G595E | Not given | Not given | III | -0.65 | -0.52 | -0.78 | -1 | -3.46 | -3.36 | -3.05 | -0.4 | **D** |
| rs370784440 | 17 | A622S | T=0.0001/1 (GoESP) | < 1% (A) | III | -1.62 | -0.69 | -0.91 | -1.20 | -2.18 | -2.32 | -2.11 | -1.04 | **D** |
| rs146219485 | 18 | I627S | G=0.0001/1 (GoESP) | < 1% (G) | III | -2.5 | -2.11 | -3.51 | -3.01 | -1.62 | -4.21 | -1.52 | -2.4 | **D** |
| rs748978616 | 18 | D628G | Not given | Not given | III | -1.39 | -1.12 | -0.5 | -0.47 | -0.51 | -0.37 | -0.64 | -1.67 | **D** |
| rs146370823 | 18 | A664V | T=0.0002/1 (1000Genomes) | < 1% (T) | III | -0.09 | -0.03 | -0.46 | -0.57 | -0.7 | 0.16 | -0.75 | -0.55 | **D** |
| rs533681648 | 19 | A689T | A=0.0002/1 (1000Genomes) | < 1% (A) | III | -1.37 | -0.58 | -1.2 | -1.55 | -1.98 | -3.04 | -1.85 | -0.79 | **D** |
| rs370858384 | 19 | T706I | T=0.0001/1 (GoESP) | < 1% (T) | III | -0.14 | -0.04 | -0.8 | -0.61 | 0.08 | -0.2 | -0.11 | 0.16 | NC |
| rs374677654 | 20 | I711T | A=0.0001/1 (GoESP) | < 1% (A) | III | -1.32 | -2.05 | -2.2 | -2.09 | -1.25 | -2.79 | -1.23 | -1.6 | **D** |
| rs767931423 | 19 | G741D | A=0.0002/43 (GnomAD_exomes) | < 1% (A) | III | -1.24 | -0.75 | -0.44 | -0.82 | -0.79 | 0.18 | -1.01 | -0.65 | **D** |
| rs750938630 | 20 | G741S | Not given | Not given | III | -1.36 | -0.90 | -0.33 | -0.73 | -0.71 | 1.04 | -1.07 | -0.79 | **D** |
| rs773394492 | 21 | G746V | Not given | Not given | III | -0.16 | 0.19 | -1.24 | -1.29 | -0.22 | 2.79 | -0.48 | -0.62 | **D** |
| rs142230684 | 21 | G746R | A=0.0002/1 (1000Genomes) | < 1% (A) | III | -0.58 | 0.19 | -0.1 | -0.65 | -1.13 | 3.91 | -1.4 | -0.8 | **D** |
| rs35217482 | 21 | T755I | T=0.0020/10 (1000Genomes) | < 1% (T) | III | -0.67 | 0.06 | -0.48 | -0.93 | -0.28 | 2.36 | -0.41 | -0.04 | **D** |
| rs141342059 | 21 | P762L | T=0.0006/3 (1000Genomes) | < 1% (T) | III | -0.32 | 0.04 | -0.52 | -0.69 | -0.46 | 2.74 | -0.79 | -0.68 | **D** |
| rs375296908 | 21 | M769T | C=0.0001/1 (GoESP) | < 1% (C) | III | -1.3 | -0.66 | -1.85 | -2.17 | -2.12 | -3.37 | -2.18 | -2.08 | **D** |
| rs765381141 | 21 | Q776V | Not given | Not given | III | 0 | -0.04 | 0.91 | 0.40 | 0.01 | 1.11 | 0.03 | -0.51 | **S** |
| rs772383712 | 22 | L789V | Not given | Not given | III | -1.24 | -1.79 | -1.41 | -1.58 | -1.75 | -1.91 | -1.44 | -1.33 | **D** |
| rs113582006 | 22 | R802H | A=0.0006/3 (1000Genomes) | < 1% (A) | III | -0.79 | -1.27 | -0.72 | -0.70 | -1.19 | 0.37 | -1.11 | -1.63 | **D** |
| rs41309768 | 22 | R802C | T=0.0001/8 (ExAC) | < 1% (T) | III | -0.58 | -1.02 | -0.62 | -0.62 | -0.35 | 0.88 | -0.32 | -1.16 | **D** |
| rs373370000 | 22 | A806V | T=0.0002/3 (GoESP) | < 1% (T) | III | -0.44 | 0.10 | -0.46 | -1.19 | -0.45 | -0.43 | -0.39 | -0.51 | **D** |
| rs777841231 | 22 | A818E | Not given | Not given | III | -1.55 | -0.56 | -0.07 | -1.26 | -2.09 | -3.14 | -1.93 | -0.68 | **D** |
| rs746205075 | 22 | A825T | A=0.0001/2 (GnomAD) | < 1% (A) | III | -1.33 | -0.62 | -0.83 | -1.31 | -2.03 | -3.04 | -1.9 | -1.27 | **D** |
| rs769159903 | 23 | R833H | Not given | Not given | III | -2.07 | -1.34 | -1.01 | -1.14 | -2.04 | -0.48 | -1.81 | -0.98 | **D** |
| rs746500211 | 23 | R838Q | A=0.0001/8 (TOPMED) | < 1% (A) | III | -0.83 | -0.83 | -1.22 | -1.37 | -1.52 | -2.24 | -1.26 | -1.01 | **D** |
| rs377368396 | 23 | D841H | C=0.0002/1 (1000Genomes) | < 1% (C) | III | 0.08 | -0.61 | -0.09 | -0.31 | -1.82 | 0.02 | -1.75 | -1.28 | **D** |
| rs762829482 | 23 | R848H | Not given | Not given | III | -0.67 | -0.97 | -1.01 | -1.28 | -2.26 | 0.26 | -1.92 | -0.9 | **D** |
| rs202238148 | 23 | P850R | G=0.0001/1 (GoESP) | < 1% (G) | III | -0.48 | -0.92 | -0.54 | -1.04 | -1.14 | -1.99 | -1.18 | -0.85 | **D** |
| rs766596324 | 24 | F859V | Not given | Not given | III | -1.3 | -1.35 | -0.7 | -1.20 | -2.93 | -0.75 | -2.74 | -1.3 | **D** |
| rs779078162 | 24 | L866W | Not given | Not given | III | -1.52 | -1.73 | -1.31 | -1.76 | -1.17 | 2.06 | -1.42 | -1.56 | **D** |
| rs369320698 | 25 | R904S | T=0.0001/3 (GnomAD) | < 1% (T) | III | -1.01 | -1.32 | -0.99 | -1.44 | -1.82 | -0.38 | -1.78 | -0.92 | **D** |
| rs374847145 | 25 | R906W | Not given | Not given | III | -0.44 | -0.37 | -0.75 | -0.60 | -0.41 | 2.49 | -0.31 | -0.8 | **N** |
| rs757648677 | 25 | N913S | Not given | Not given | III | -0.56 | -0.53 | -0.34 | -0.62 | -1.61 | -0.85 | -1.59 | -1.37 | **D** |
| rs753249922 | 25 | L914R | Not given | Not given | III | -1.63 | -1.77 | -0.49 | -1.33 | -0.93 | -3.07 | -0.91 | -1.81 | **D** |
| rs765938790 | 25 | L914F | Not given | Not given | III | -1.09 | -1.26 | -0.37 | -0.87 | -1.63 | 0.54 | -1.48 | -1.13 | **D** |
| rs56199635 | 25 | R921H | Not given | Not given | III | -0.76 | -1.11 | -0.7 | -1.04 | -2.38 | 0.26 | -2.16 | -1.02 | **D** |
| rs144275574 | 25 | G924A | C=0.0001/1 (GoESP) | < 1% (C) | III | -0.62 | -0.64 | -1.02 | -0.88 | -1.05 | -1.12 | -0.84 | -0.93 | **D** |
| rs142604856 | 25 | E933K | A=0.0002/1 (1000Genomes) | < 1% (A) | III | -1.12 | -0.4 | -0.38 | -0.31 | -0.77 | -3.27 | -0.64 | -1.35 | **D** |
| rs142541240 | 26 | R951Q | A=0.0002/1 (1000Genomes) | < 1% (A) | III | -1.75 | -1.2 | -1.28 | -1.14 | -1.34 | -0.63 | -1.33 | -0.57 | **D** |
| rs780973244 | 26 | Y956C | Not given | Not given | III | -1.75 | -0.95 | -1.52 | -2.18 | -1.77 | 0.03 | -1.81 | -1.24 | **D** |
| rs138174107 | 26 | Q966K | T=0.0006/3 (1000Genomes) | < 1% (T) | III | -0.62 | -0.14 | -0.56 | -0.95 | -0.49 | -1.08 | -0.45 | -0.87 | **D** |
| rs776783393 | 26 | A1008T | A=0.0001/9 (ExAC) | < 1% (A) | III | -1.24 | -0.51 | -0.58 | -1.09 | -1.6 | -0.52 | -1.76 | -1.29 | **D** |
| rs747923718 | 25 | A1026T | Not given | Not given | III | -0.85 | -0.61 | -0.4 | -0.69 | -1.58 | -2.16 | -1.57 | -0.3 | **D** |
| rs544496025 | 27 | A1028P | C=0.0004/2 (1000Genomes) | < 1% (C) | III | -1.06 | -0.11 | -1.82 | -2.07 | -0.9 | -3.35 | -0.69 | -1.5 | **D** |
| rs772711058 | 27 | A1028V | T=0.0001/11 (ExAC) | < 1% (T) | III | -0.24 | 0.01 | -0.26 | -0.46 | -0.42 | 1.02 | -0.69 | -0.99 | **D** |
| rs765106050 | 27 | Y1033C | G=0.0000/2 (ExAC) | < 1% (G) | III | -1.29 | -0.90 | -1.5 | -1.52 | -0.67 | 2.06 | -0.84 | -1.17 | **D** |
| rs530086359 | 27 | H1042P | G=0.0002/1 (1000Genomes) | < 1% (G) | III | -0.93 | 0.28 | -0.97 | -1.44 | -0.86 | -3.25 | -0.51 | -1.12 | **D** |
| rs747686137 | 27 | T1053I | Not given | Not given | III | -0.3 | -0.09 | -0.83 | -0.87 | 0.34 | 2.58 | 0.09 | -0.08 | **S** |
| rs373988743 | 27 | K1054N | T=0.0001/1 (GoESP) | < 1% (T) | III | -1.29 | -0.32 | -0.97 | -1.16 | -1.18 | 0.83 | -1.2 | -0.26 | **D** |
| rs759347833 | 28 | S1060R | G=0.0002/1 (Estonian) | < 1% (G) | III | -0.94 | -0.05 | -0.02 | -0.65 | -0.99 | -0.89 | -1.11 | -0.45 | **D** |
| rs531382479 | 28 | S1089P | C=0.0002/1 (1000Genomes) | < 1% (C) | III | -0.75 | -0.43 | -0.73 | -1.26 | -1.28 | -8.6 | -0.5 | -0.72 | **D** |
| rs139975106 | 28 | G1096S | A=0.0002/1 (1000Genomes) | < 1% (A) | III | -1.33 | -1.31 | -0.75 | -0.91 | -1.94 | -0.55 | -2.04 | -0.67 | **D** |
| rs540086318 | 29 | A1102D | A=0.0002/1 (1000Genomes) | < 1% (A) | III | -1.54 | -0.56 | -1.77 | -2.14 | -3.48 | -3.65 | -3.25 | -0.5 | **D** |
| rs750075479 | 29 | R1109C | Not given | Not given | III | -0.7 | -0.84 | -0.62 | -0.50 | -0.72 | 1.11 | -0.83 | -0.88 | **D** |
| rs376172931 | 29 | R1109H | T=0.0002/1 (1000Genomes) | < 1% (T) | III | -1.15 | -1.18 | -0.93 | -0.80 | -1.59 | 0.4 | -1.58 | -1.16 | **D** |
| rs201601639 | 30 | F1142L | Not given | Not given | III | -0.18 | -1.31 | -1 | -1.63 | -0.96 | -0.18 | -0.88 | -0.44 | **D** |
| rs148902105 | 31 | Y1164C | G=0.0002/1 (1000Genomes) | < 1% (G) | III | -1.71 | -0.86 | -1.5 | -1.81 | -1.95 | 1.65 | -1.89 | -0.35 | **D** |
| rs35345784 | 31 | A1167V | Not given | Not given | III | -0.45 | -0.09 | 0.22 | -0.36 | 0.03 | 1.02 | -0.28 | -0.45 | NC |
| rs143571444 | 31 | S1169Y | A=0.0002/1 (1000Genomes) | < 1% (A) | III | -0.69 | 0.08 | 0.13 | -0.19 | -0.55 | 2.12 | -0.57 | -0.33 | **D** |
| rs769549935 | 31 | E1170K | Not given | Not given | III | -1.39 | -0.83 | -0.68 | -1 | -0.94 | -1.15 | -0.96 | -0.79 | **D** |
| rs773323708 | 31 | E1170R | Not given | Not given | III | -0.85 | -0.21 | -0.32 | -0.81 | -0.71 | -0.18 | -0.87 | -0.53 | **D** |
| rs779495901 | 31 | G1178R | A=0.0002/5 (GnomAD) | < 1% (A) | III | -1.06 | -0.63 | -0.59 | -0.63 | -1.32 | -1.64 | -1.35 | -0.85 | **D** |
| rs369814413 | 32 | S1194N | A=0.0002/19 (ExAC) | < 1% (A) | III | -1.57 | -0.53 | -0.57 | -0.67 | -0.95 | 1.23 | -1.02 | -0.51 | **N** |
| rs368816835 | 33 | G1213E | A=0.0002/2 (GoESP) | < 1% (A) | III | -1.04 | -0.47 | -1.1 | -1.1 | -2.41 | 0.33 | -2.49 | -0.26 | **D** |
| rs150568828 | 33 | Y1215H | C=0.0002/1 (1000Genomes) | < 1% (C) | III | -1.26 | -1.22 | -1.07 | -1.25 | -2.05 | -0.95 | -1.97 | -0.94 | **D** |
| rs768035591 | 33 | L1228R | Not given | Not given | III | -1.47 | -1.67 | -1.28 | -1.55 | -0.74 | -2.5 | -0.7 | -1.48 | **D** |
| rs373230627 | 33 | R1231H | A=0.0001/1 (GoESP) | < 1% (A) | III | -0.66 | -1.08 | -0.04 | -0.35 | -0.63 | 0.04 | -0.6 | -1.51 | **D** |
| rs766738403 | 33 | K1237N | Not given | Not given | III | -1.13 | -0.4 | -0.9 | -1.38 | -1.46 | 1.13 | -1.43 | -0.19 | **D** |
| rs374349831 | 33 | P1245L | T=0.0001/1 (GoESP) | < 1% (T) | III | -1.02 | -0.35 | -1.04 | -0.66 | -0.74 | 0.92 | -0.91 | 0.47 | **D** |
| rs774474413 | 33 | L1262H | Not given | Not given | III | -2.43 | -1.82 | -0.81 | -1.48 | -2.04 | -2.47 | -1.79 | -2.52 | **D** |
| rs143420132 | 34 | G1269R | Not given | Not given | III | -0.76 | -0.52 | -0.19 | -0.70 | -0.85 | -1.64 | -0.76 | -0.17 | **D** |
| rs141786030 | 34 | S1271L | T=0.0002/2 (GoESP) | < 1% (T) | III | -0.36 | -0.27 | 1 | 1.16 | 0.74 | 1.19 | 0.6 | 0.12 | **S** |
| rs267599151 | 34 | S1278F | Not given | Not given | III | -0.19 | -0.04 | -0.56 | -0.41 | -0.96 | 3.03 | -0.9 | -0.26 | **D** |
| rs777157791 | 34 | V1279M | Not given | Not given | III | -0.86 | -1.19 | -0.69 | -1.13 | -1.23 | -0.08 | -1.13 | -0.52 | **D** |
| rs139412477 | 34 | F1281L | C=0.0002/1 (1000Genomes) | < 1% (C) | III | -0.87 | -1.01 | -1.37 | -1.65 | -1.41 | -0.17 | -1.39 | -0.91 | **D** |
| rs150828500 | 34 | R1291P | C=0.0001/1 (GoESP) | < 1% (C) | III | -0.92 | -0.44 | -1.33 | -1.72 | -1.37 | -1.74 | -1.08 | -1.02 | **D** |
| rs771024005 | 34 | T1307I | Not given | Not given | III | -0.18 | -0.27 | -0.85 | -1.15 | -0.13 | 1.63 | 0 | -0.25 | **D** |
| rs375761698 | 35 | P1324R | T=0.0001/1 (GoESP) | < 1% (T) | III | -1.01 | -0.91 | 0.41 | -0.04 | 0.09 | -0.82 | -0.08 | -0.94 | **D** |
